# The Army Bill

**Published:** 1898-08

**Authors:** 


Every veterinary organization at its opening fall meeting should take up the question of army legislation and plan to aid the national organization during the coming year in securing a reorganization of our army veterinary service. The time is opportune, the need never more forcibly presented, and the hour to wipe away this reflection upon our profession has arrived. It can only be done by a strong united effort and a good active representation at Washington, that will be properly supported by the veterinary profession.

